# Dutch Validation of the Self-Evaluation of Negative Symptoms Scale (SNS)

**DOI:** 10.3390/brainsci15010015

**Published:** 2024-12-27

**Authors:** Tim van Brouwershaven, Anika Poppe, Gerdina Hendrika Maria Pijnenborg, André Aleman, Nynke Boonstra, Shiral Gangadin, Sonia Dollfus, Wim Veling, Stynke Castelein, Jan Alexander de Vos, Edith Liemburg, PHAMOUS-researchers, Lisette van der Meer

**Affiliations:** 1 Department of Rehabilitation, Lentis Psychiatric Institute, 9471 KA Zuidlaren, The Netherlands; t.vanbrouwershaven1@lentis.nl (T.v.B.);; 2 Department of Clinical and Developmental Neuropsychology, University of Groningen, 9712 TS Groningen, The Netherlands; 3 Department of Psychotic Disorders, GGZ Drenthe, 9404 LA Assen, The Netherlands; 4 Faculty of Psychology and Neuroscience, Maastricht University, 6211 LK Maastricht, The Netherlands; 5 Department of Neuroscience, UMC Utrecht, 3584 CG Utrecht, The Netherlands; 6 Department of Research and Innovation, KieN VIP Mental Health Care Services, 8911 KJ Leeuwarden, The Netherlands; 7 Department of Psychiatry, UMC Groningen, 9713 GZ Groningen, The Netherlands; 8 Physiopathology and Imaging of Neurological Disorders, UMR S 1237, GIP Cyceron, 14032 Caen, France; 9 Fédération Hospitalo-Universitaire (FHU A2M2P), CHU de Caen Normandie, 14000 Caen, France; 10 UFR de Santé, Université de Caen Normandie, 14000 Caen, France; 11 Department of Psychology, University of Twente, 7522 NB Enschede, The Netherlands; 12 Department of Research, GGZ Friesland Mental Healthcare Institution, 8901 BS Leeuwarden, The Netherlands

**Keywords:** schizophrenia, negative symptoms, scales, validation, self-assessment, psychosocial functioning, quality of life

## Abstract

Background/objectives: Negative symptoms in schizophrenia spectrum disorders are related to impaired social functioning and lower quality of life, making accurate assessment important. To date, most tools for assessing negative symptoms are observational, which can be influenced by the raters’ experience and opinion. Self-rating scales, like the Self-Evaluation of Negative Symptoms (SNS), could complement observer ratings by adding information from the patient’s perspective. Here, we aim to evaluate the psychometric properties of the Dutch translation of the SNS and the relationship between the SNS and functional outcomes. Methods: The SNS was added to the Pharmacotherapy Monitoring Outcome Survey (PHAMOUS)-protocol for adults with a DSM-5 classification of a disorder in the psychosis spectrum. Internal consistency was assessed by Cronbach’s alpha. Confirmatory factor analysis (CFA) was used to evaluate the construct validity of the five subscales of the SNS. Correlational analyses were performed between the SNS and the Positive and Negative Syndrome Scale (PANSS), the Health of Nation Outcomes Scales (HoNOS), the Global Assessment of Functioning (GAF), Functional Remission tool (FR) and the Manchester Short Assessment of Quality of Life (ManSA). Results: A total of 247 patients participated in this study. Internal consistency was good (α = 0.87). CFA confirmed the five-factor structure of the SNS. The SNS was significantly correlated (all *p* < 0.001) with the PANSS positive (r = 0.31), PANSS negative (r = 0.33), HoNOS (r = 0.37), FR (r = 0.27) and the ManSA (r = −0.40). Conclusions: The Dutch SNS shows good psychometric properties and is related to functional outcomes and quality of life. The SNS can be valuable in complementing current observational-based instruments, and future research may investigate whether the SNS can be used as a standalone measurement tool for the assessment of negative symptoms.

## 1. Introduction

Negative symptoms have been regarded as core features of schizophrenia spectrum disorders (SSDs) for a long time and are persistently present in 35–70% of the people with a classification in this spectrum [1,2,3,4,5]. They are considered a major burden and are related to impaired global and social functioning [6,7] and a lower quality of life [8]. Furthermore, negative symptoms are often present during the prodromal phase of SSD and have been characterized as risk factors for developing psychosis [9,10,11]. Therefore, accurate treatment of these negative symptoms is important, but remains challenging. To date, limited effective treatments for negative symptoms are available [12,13].

In 2006, the National Institute of Mental Health (NIMH) initiated a Consensus meeting to examine problems that may interfere with treatment development. Here, it was agreed upon that negative symptoms are considered a distinct therapeutic area, and that factor analyses support five domains: social withdrawal, blunted affect (or reduced emotional range), alogia, avolition and anhedonia [14]. Widely used assessment tools, such as the Positive and Negative Syndrome Scale (PANSS) [15], the Scale for the Assessment of Negative Symptoms (SANS) [16], the Brief Negative Symptom Scale (BNSS) [17] and the Clinical Assessment Interview for Negative Symptoms (CAINS) [18], evaluate negative symptom severity based on ratings by an interviewer and can thus be influenced by the raters’ experience and opinion [19]. Clinician ratings are important since people who experience symptoms are not always capable of recognizing their symptoms [20,21,22], and the personally experienced burden of negative symptoms may differ from observation by the raters. In addition, while people with schizophrenia often express positive and negative emotions to a lesser extent than healthy controls, they experience the same range of these emotions [23,24,25]. Self-rating scales could complement observer-based measurements by including information from the patient’s perspective, which is particularly important as these scales often capture inner, non-observable outcomes that may be less visible to medical staff or caregivers [26]. Furthermore, self-rating scales allow people with an SSD classification to evaluate their functioning and self-reflect on their symptoms. This may improve illness insight and, thereby, improve their involvement and commitment to the therapeutic process [26].

To this end, Dollfus et al. [27] created the Self-Evaluation of Negative Symptoms (SNS), a self-assessment scale for negative symptoms that can be completed in five minutes. The SNS was developed in French and has been translated into 27 languages so far, including English [27], Polish [28], Spanish [29], Arabic [30], Lithuanian [31], Chinese [32], Persian [33] and Turkish [34]. Recently, a large European and multicenter study demonstrated that the SNS has adequate psychometric properties and an association with functional outcomes (as measured with the Personal and Social Performance Scale (PSP)) [35]. In addition, the SNS has demonstrated its validity in screening negative symptoms in subjects with first psychotic episode [36].

The SNS has not been translated to Dutch before. Here, we aim to evaluate the psychometric properties (internal consistency and the construct, convergent, and discriminant validity) of the Dutch translation of the SNS and the relationship between self-rated negative symptoms, observer-rated negative symptoms, functional outcomes and quality of life in patients with SSD.

## 2. Materials and Methods

### 2.1. Study Design and Participants

Between 2020 and 2022, the SNS was added to the Pharmacotherapy Monitoring Outcome Survey (PHAMOUS)-protocol. This is an annual screening of mental and physical health of people receiving care in one of the four participating mental health institutions in the North of the Netherlands as a part of regular clinical practice [37]. Data were requested from individuals who (1) have a DSM-5 classification of a psychotic disorder and (2) are 18 years or older. The minimal required sample size of 175 people was based on the recommendation of the COnsensus-based Standards for the selection of health Measurement Instruments (COSMIN) [38]: the number of items of the SNS times was seven after taking into account 25% of missing data. The following demographics were assessed: age, gender, education and daily activities. Education was categorized as lower (no education, primary school or prevocational education), middle (secondary vocational education) and higher education (tertiary vocational education). In accordance with the declaration of Helsinki, patients were informed that aggregated and anonymized data may be used for healthcare optimization and scientific research to improve treatment and guidance [37].

### 2.2. Measurement Instruments

#### 2.2.1. SNS

The Self-Evaluation of Negative Symptoms (SNS) includes five domains of negative symptoms (i.e., social withdrawal, blunted affect, alogia, avolition and anhedonia) and contains 20 items: 4 items for each domain. There are three possible responses, “strongly agree”, “somewhat agree” and “strongly disagree”, and the total score ranges from 0 to 40, where a higher score indicates more severe symptoms.

The Dutch version was translated from the English version [27] and was thereafter back-translated into French. Discrepancies were discussed with the scale author (Prof. Sonia Dollfus), upon which Prof. Dollfus approved the Dutch translation.

#### 2.2.2. PANSS

Symptom severity was assessed by using the Positive and Negative Syndrome Scale (PANSS), a widely used 30-item semistructured interview with high internal consistency and good construct validity [15]. The PANSS evaluates the presence and severity of positive and negative symptoms and general psychopathology within the past week. The scale consists of seven items regarding positive symptoms, seven for negative symptoms and sixteen for general psychopathology. Rating is based on a seven-point scale, and the total score ranges from 30 to 210, where higher scores indicate more severe symptoms [15].

#### 2.2.3. HoNOS

The Health of the Nation Outcome Scale (HoNOS) is an observational scale consisting of twelve items measuring social and general functioning in the past two weeks. The HoNOS consists of four domains: behavioral problems, impairment, symptomatology and social problems. Each item is rated on a five-point scale ranging from ‘no problem’ to ‘severe to very severe problem’ where higher scores indicate more severe problems [39].

#### 2.2.4. GAF

The Global Assessment of Functioning (GAF) is a numeric observational scale ranging from 0 to 100, which measures global functioning. The level of the psychological, social and occupational functioning of the individual integrates within a single score of global functioning rated by a clinician. A high score indicates better outcomes [40].

#### 2.2.5. Functional Remission

This observational scale is an indication of functional remission in several domains: (1) daily living and self-care, (2) work, study and housekeeping and (3) social contacts. These domains are rated on a three-point scale by a clinician (absence of the problem, presence of the problem that is marginal or covered by support, presence of the problem that is insufficiently covered by support), where lower scores indicate more remission [41].

#### 2.2.6. MANSA

The Manchester Short Assessment of Quality of Life (MANSA) is a self-report questionnaire to assess quality of life. It addresses patients’ satisfaction within several psychosocial domains, including satisfaction with life as a whole, job (or unemployment/retirement), financial situation, number and quality of friendships, leisure activities, accommodation, physical and mental health and safety. The twelve items rated on a 7-point scale, ranging from “couldn’t be worse” to “couldn’t be better” were used for analysis (four items are dichotomous (yes/no) and were excluded for methodological reasons). Total scores range from 12 to 84, and higher scores indicate higher reported quality of life.

### 2.3. Statistical Analysis

For the internal consistency of the total score and the five subscales of the SNS, Cronbach’s alpha was calculated, where values ≥ 0.7 are considered acceptable [42]. Furthermore, we calculated ‘alpha if item deleted’; Cronbach’s alpha was calculated when any of the items were dropped from the SNS, to test whether the deletion of any question would influence the reliability of the (sub)scale. The construct validity of the five-factor structure was evaluated through confirmatory factor analysis (CFA). Social withdrawal, reduced emotional range, alogia, avolition and anhedonia subscores were entered as latent variables. To measure the goodness-of-fit (GOF) of the factor structure, the following criteria were used: the Comparative Fit Index (CFI), the Tucker–Lewis Index (TLI) and the Root Mean Square Error of Approximation (RMSEA). An acceptable fit of the model is suggested by RMSEA values < 0.06 and CFI and TLI values > 0.9 [43]. Correlational analyses between the SNS and PANSS negative subscore was performed to assess convergent validity. Discriminant validity was calculated using the correlation coefficient between the SNS and the PANSS positive subscore. To evaluate the relationship between self-rated negative symptoms, observer-based negative symptoms and functional outcomes, correlational analyses between the SNS, the PANSS negative subscale, the HoNOS total score, GAF, functional remission total score and the ManSA total score were performed. To assess whether correlation coefficients of SNS subdomains with outcomes were statistically different, Fisher Z-transformation was performed. Data were not normally distributed, and therefore, Spearman’s rho correlations were used to check for associations between the different outcome variables. A *p*-value < 0.05 was considered significant. However, *p*-values are generally highly significant even for very low correlation coefficients with large sample sizes (N > 200). Therefore, the absolute value of the correlation coefficient is an even more meaningful estimation of the association than with smaller sample sizes. Statistical analysis was performed using R version 4.3.1. [44] by using the ‘psych’ [45] (internal consistency), ‘lavaan’ [46] (confirmatory factor analysis) and ‘naniar’ [47] (handle missing data) packages. If missing data were <25% of a (sub)domain of an outcome variable, mean imputation of this domain was applied to address missing data in our sample (Supplementary Data).

## 3. Results

### 3.1. Demographics

In total, 247 people were included in this study. The mean age was 42.0 ± 12.5 years, and 34.4% (N = 85) was female. Data on demographics and outcome variables are provided in Table 1.

### 3.2. Psychometric Properties

#### 3.2.1. Internal Consistency

The Cronbach’s alpha coefficient of the SNS total scale was 0.87. The coefficients for the different subscales varied between 0.64 and 0.79 (Table 2). Withdrawal of any question did not lead to a higher coefficient for the total scale. In the different subscales, only the withdrawal of items 12 (With friends and family, I want to talk about things but it doesn’t come out) and 20 (I am not interested in having sex) resulted in an increase in the value of Cronbach’s alpha in the alogia (0.71 vs. 0.72) and anhedonia (0.69 vs. 0.72) subscales, respectively.

Furthermore, the intercorrelations of the SNS subscores were all highly significant, ranging from 0.26 to 0.52 (Table 3).

#### 3.2.2. Confirmatory Factor Analysis

The good fit of a five-factor model is supported by TLI and CFI > 0.9 and RMSEA < 0.06. The CFI (0.919) and the TLI (0.904) as well as the RMSEA (0.054) showed an adequate fit for the five-factor structure of the SNS.

#### 3.2.3. Convergent and Discriminant Validity

Correlational analyses between the SNS and the PANSS negative and positive subscale were performed to assess convergent and discriminant validity, respectively. Regarding convergent validity, the total score of the SNS showed a significant but modest positive correlation with the PANSS negative symptom subscale (r = 0.33; *p* < 0.001) (Table 4). For discriminant validity, the total score of the SNS resulted in a significant positive modest correlation of r = 0.31 (*p* < 0.001) with the PANSS positive subscale (Table 4).

A total of 61.5% (N = 152) of the people did not score a three or higher (mild symptoms) in any of the seven positive subscale items. To examine to which extent the absence of symptoms influenced the corelation coefficient, a post hoc analysis was performed with a subset of people who scored a three or higher in any of the seven positive subscale items (n = 95). This resulted in a weak non-significant correlation between the SNS total and the PANSS positive subscale (r = 0.066; *p* = 0.52) (Table 5). Further, the correlation coefficient between the SNS and the PANSS negative subscale remains highly significant.

### 3.3. Functional Outcomes

The total score of the SNS showed a significant, moderate positive correlation with the HoNOS (r = 0.37; *p* < 0.001) and a significant weak correlation with FR (r = 0.27; *p* < 0.001) (Table 4). The negative correlation between the SNS total score and the GAF was weak and not significant (r = −0.12; *p* = 0.16). The PANSS negative subscale was significantly correlated with the HoNOS (r = 0.44; *p* < 0.001), FR (r = 0.53; *p* < 0.001), and the GAF (r = −0.43; *p* < 0.001) (Table 4). In the different subscales, the correlation coefficient of reduced emotional range was significantly lower than social withdrawal (*p* = 0.045) and avolition (*p* = 0.026) in the HoNOS, and significantly lower than social withdrawal (*p* = 0.016) and avolition (*p* = 0.044) in the FR (Supplementary Data). The correlation coefficient of alogia was significantly lower than social withdrawal (*p* = 0.016) and almost significantly lower than avolition (*p* = 0.050) in the FR (Supplementary Data).

### 3.4. Quality of Life

The total score of the SNS showed a significant negative moderate correlation with the self-report questionnaire MANSA (r = −0.40; *p* < 0.001) (Table 4). The negative subscale of the PANSS showed a significant negative moderate correlation with the MANSA (r = −0.35; *p* < 0.001) (Table 4). In the different subscales, the negative correlation coefficient of avolition was significantly higher than social withdrawal (*p* < 0.001), reduced emotional range (*p* < 0.001), and alogia (*p* = 0.0080) (Supplementary Data). The negative correlation coefficient of anhedonia was significantly higher than social withdrawal (*p* = 0.025) and reduced emotional range (*p* = 0.0010) (Supplementary Data).

## 4. Discussion

Negative symptoms have been regarded as a core feature of SSD and represent an unmet treatment need. Accurate assessment tools that capture the internal experience of people suffering from SSD are highly important. Therefore, we examined the reliability, validity and associations with functional outcomes of the Dutch translation of the SNS as a self-assessment tool for negative symptoms. Our findings indicate that the SNS has good internal consistency, construct validity and discriminant validity, and modest convergent validity, and is related to functional outcome and quality of life.

### 4.1. Psychometric Properties

#### 4.1.1. Internal Consistency

Reliability analysis showed good internal consistency of the SNS, similar to those of the French [27], Arabic [30], Polish [28], Spanish [29], Turkish [34] and Lithuanian [31] versions, ranging from 0.82 to 0.97. Similarly, looking at the subscales individually (i.e., social withdrawal, alogia, avolition, reduced emotional range and anhedonia), we found good internal consistency, though Cronbach’s alpha for the subscales reduced emotional range and anhedonia was moderate. Two prior SNS validation studies examined the reliability of the individual subscales and found similar results: the Turkish and Spanish version of the SNS reported a lower value of the reduced emotional range subscale compared to the other subscales (α = 0.523 and 0.59, respectively) [29,34]. The reliability may be affected by items 5 (People say I’m not sad or happy and that I’m not often angry) and 8 (it is difficult for people to know how I feel), since these items depend on observation of others and on Theory of Mind (the ability to understand others’ beliefs, emotions, and thoughts), which is often impaired in people with psychotic disorders [48,49]. Next, we found moderate reliability for the anhedonia subscale, again in line with the Turkish and Spanish translation (α = 0.69 and 0.61, respectively). Interestingly, the deletion of item 20 (I am not interested in having sex) increased the value of Cronbach’s alpha. It is possible that other factors also play a role in a decreased interest in sex. Reduced interest in sexual activities may, next to the limited ability to experience pleasure, result from emotional, motivational, and social factors [50], as well as the limited opportunity to engage in sexual relationships, which could explain the increase in reliability of the anhedonia scale after removing this item. Next, the deletion of item 12 (With friends and family, I want to talk about things, but it doesn’t come out) increased the reliability of the alogia scale. However, the subscale including this item still showed adequate reliability and the effect of deleting the question was considered to be minimal and therefore was not removed from the questionnaire. Taken together, despite the moderate reliability of reduced emotional range and anhedonia, significant correlations with the other subscales and good construct validity of the total scale suggest that the factors are valid and confirm the importance of these five subdomains and questions.

#### 4.1.2. Construct Validity

For the construct validity, CFA confirmed the five-factor structure of the SNS. The different scores indicated a good fit, as described in the literature [43]. Together with the Turkish [34], Spanish [29] and Chinese [32] versions and a large European multicenter study [35], our results show good construct validity. This suggests that the SNS is able to distinguish between the five subdomains of negative symptoms and can produce meaningful subscores for each domain. Future research may use the SNS to evaluate the effect of interventions and therapeutic agents on each subdomain individually rather than the effect on negative symptoms as a whole.

### 4.2. Convergent and Discriminant Validity

For convergent validity, we examined the correlation between the SNS and the PANSS negative subscale. The total score and the different subscores of the SNS were all significantly associated with the PANSS negative subscale. However, the correlation coefficient was modest (r = 0.33). This is consistent with a previous study from several European centers [35], that reported a correlation of 0.37. Possibly, the SNS is a more specific and elaborate measure of negative symptoms than the PANSS, which could contribute to the limited correlation, i.e., there is no full overlap. In addition, the difference in perspective between the clinician and the participant could help explain this modest correlation. The literature suggests that people with schizophrenia experience a similar range of positive and negative emotions as healthy controls, but fail to express these feelings [23,24,25]. This discrepancy in what clinicians report about emotional expressions and what people with schizophrenia actually experience could explain this modest association we found in this study. If so, it would mean that the SNS is able to add information about the internal experience that is lacking from the PANSS. Alternatively, it is possible that self-reporting negative symptoms is particularly challenging for some individuals. While discrepancies between expressed and experienced emotions have been documented, some participants may struggle to recognize or accurately identify their own symptoms. This difficulty in self-awareness could partly explain the low correlation observed between the PANSS negative symptom scale and the SNS. In both scenarios, the self-experience of negative symptoms could offer valuable insights for clinicians, enhancing their understanding and informing treatment approaches.

For discriminant validity, the correlation between the SNS and the PANSS positive subscale was investigated. The significant modest correlation (r = 0.31; *p* < 0.001) we found was higher compared to other translations: the Arabic (r = −0.11; *p* = 0.53) [30], Turkish (r = 0.14; *p* = 0.90) [34] and European multicenter validation study (r = 0.12; *p* = non-significant) [35] all reported weak non-significant correlations between the total SNS score and the PANSS positive subscale. This may be due to the relatively low mean PANSS positive subscale and SNS scores of our sample (9.79 ± 3.18). Both the SNS scores [27,30,31,33,34,35] and the PANSS positive subscale [34,35] of our sample were the lowest compared to other translations, with 37.68% (N = 78) scoring minimal in the positive domain. It may be that the absence of symptoms influenced the correlation coefficient, so to examine to what extent this was true, a post hoc analysis with a subgroup of people with mild positive symptoms in at least one domain was performed. This resulted in a weak non-significant correlation, which is in line with the other translations, suggesting that the relatively low scores may have affected the discriminant validity of the total sample. Future research in a more heterogeneous sample may be needed to confirm this.

### 4.3. Functioning and Quality of Life

As previously stated, negative symptoms are related to impaired global and social functioning [6,7]. Here, the total score of the SNS was significantly correlated with the HoNOS and the FR. Interestingly, the correlation between PANSS negative subscale and the HoNOS, GAF, and the FR was stronger than the correlation between the SNS and these instruments. Like the difference in emotional expression, this may be due to the discrepancy between observational measurement tools and self-report questionnaires.

In the different subscales, the strongest correlations in both the FR and the HoNOS are reported in the social withdrawal and avolition domains. This is in line with prior studies, which reported that *avolition*, defined as the reduced initiation and persistence of goal-directed activity due to reduced motivation [51], is a great determinant of social functioning [52,53,54,55,56]. Additionally, the European SNS validation study found that avolition (together with the PANSS positive subscores, level of education, and the lack of judgment and insight PANSS items) explained 23,4% of the variance of functional outcome [35]. Noteworthy, although the correlation coefficients of social withdrawal and avolition were the strongest, they were not significantly different from some of the subdomains. Further research may be needed to confirm that social withdrawal and avolition are suitable predictors of functioning. Additionally, reduced emotional range and alogia showed the weakest correlation with the HoNOS, and were not significantly associated with the FR. In the HoNOS, the correlation coefficient of reduced emotional range was significantly lower compared to social withdrawal and avolition. In the FR, the correlation coefficient of reduced emotional range was significantly lower than social withdrawal and avolition, and the correlation coefficient of alogia was significantly lower than social withdrawal and almost significantly lower than avolition (*p* = 0.050). The literature suggests that alogia and blunted affect have similar underpinnings and are frequently clustered together in an ‘expressive deficits factor’, which reflects a reduced emotional expression and a reduced verbal output [57,58]. Several studies reported that expressive deficits are a poor predictor of functional outcome [55,59,60,61,62,63]. Our results support these findings, and this, together with the results from the CFA, suggests that the SNS is able to successfully differentiate between the motivational and expressive domains of negative symptoms and capture these constructs by measuring the internal experience.

For quality of life, we found that the SNS is negatively correlated with the MANSA, supporting the relationship between negative symptoms and quality of life. This is in agreement with a study that found an association between the SNS and health-related quality of life [64]. Moreover, they found that in the different subdomains, avolition was the most predictive of reduced health-related quality of life. Here, in agreement with these results, avolition showed the strongest negative correlation with the MANSA. The correlation coefficient of avolition was significantly higher than social withdrawal, reduced emotional range and alogia, and almost reached significance compared to anhedonia (*p* = 0.059). Avolition has been proposed as an important factor for determining social functioning, and this indicates that it additionally may be an important factor in determining quality of life. However, the exact mechanisms underlying the relationship between avolition, functioning and quality of life remain unclear. Future research may look into this mechanism. Next, compared to the association between the SNS and the MANSA, the correlation coefficient between the PANSS negative score and the MANSA was less robust. However, the difference in correlations between the SNS and the PANSS negative score with the MANSA was small. Possibly, together with the moderate correlation between the SNS and the PANSS negative subscale, and the differences in relationships between these scales and functional outcome scales, self-report questionnaires can add valuable information which is difficult to detect with observer-based measurement tools. However, since the difference in correlation coefficients was small, a replication in a more heterogeneous sample may be needed to confirm this hypothesis.

### 4.4. Strengths and Limitations

Some strengths can be reported. First, to the best of our knowledge, this is the first SNS validation to extensively evaluate the correlation with both functional outcomes and quality of life. Second, this study focused on the differences between self-report and observer-based measurements in terms of the relationship with functional outcome and quality of life. Third, the large sample size of this study can be considered a strength. Compared to other SNS validation studies, this study included the largest sample. There are also some limitations in our study. First, the lack of heterogeneity in symptom severity is a drawback. The PANSS total score and subscores are relatively low in our sample compared to other studies and are highly intercorrelated. Second, the use of a subgroup with mild positive symptoms to establish discriminant validity can be considered a limitation. However, since we wanted to investigate to what extent the PANSS positive subscore and the SNS measure different constructs, it was decided to exclude the influence of symptom absence. Third, during the period that the SNS was added to the PHAMOUS-protocol, there was no self-report questionnaire measuring functional outcomes incorporated in the protocol, and therefore, we were not able to examine the relationship between the SNS and experienced functioning. However, since self-assessment of functioning also captures internal experience, it is likely that the SNS will show significant associations with these assessment tools. Future research may be needed to confirm this hypothesis.

## 5. Conclusions

In conclusion, the Dutch translation of the SNS shows good psychometric properties and is associated with functional outcomes and quality of life, suggesting that more self-reported negative symptoms are related to lower levels of functioning and reported quality of life. Despite the importance of accurate assessment of negative symptoms, current observational measurement tools do not include the internal experience of people suffering from SSD. The SNS is able to differentiate between the different NIMH domains, adds information from the patients’ perspective, can be completed in approximately 10 min and requires little help from the caregivers, suggesting that it can be meaningful to complement current assessment tools. Furthermore, this could make the SNS useful in the research setting, since specific training in conducting interviews and the administration of instruments like the PANSS, SANSS, BNSS, and the CAINS can be time-consuming, which can cause time issues in conducting scientific research. However, a replication in a heterogeneous sample is needed to establish if the SNS is suitable as a standalone measurement tool for negative symptoms. If so, the SNS could be a promising instrument for the focus on the effect of interventions and therapeutics on the individual domains of negative symptoms.

## Figures and Tables

**Table 1:** Demographics.

	Mean (SD)	% (N)
Age	42.9 (12.5)	
Gender (Female)		34.4 (85)
Diagnosis		
Schizophrenia		37.80 (93)
Schizoaffective disorder		11.38 (28)
Bipolar disorder		6.91 (17)
Other psychotic disorder		44.31 (109)
Education ^1^		
Lower		20.65 (51)
Middle		51.82 (128)
Higher		26.32 (65)
Daily activities		
Job		27.53 (68)
Volunteering		21.05 (52)
Day care		19.43 (48)
Household		12.15 (30)
Study		8.10 (20)
Family care		1.21 (3)
None		23.48 (58)
SNS total	12.6 (7.43)	
Social withdrawal	2.42 (2.03)	
Reduced emotional range	2.79 (1.95)	
Alogia	2.49 (2.02)	
Avolition	2.83 (2.24)	
Anhedonia	2.11 (1.86)	
PANSS total ^2^	40.4 (10.3)	
Positive subscale	9.79 (3.18)	
Negative subscale	9.57 (3.73)	
General subscale	21.0 (5.07)	
HoNOS ^3^	6.60 (5.06)	
GAF ^4^	57.0 (11.1)	
FR ^5^	2.42 (1.67)	
MANSA ^6^	59.3 (12.0)	

^1^: missing 3, ^2^: missing 40, ^3^: missing 80, ^4^: missing 113, ^5^: missing 49, ^6^: missing 65.

**Table 2:** Reliability of the SNS and the different subscales.

	Cronbach’s Alpha [CI 95%]
SNS total	0.87 [0.85–0.90]
Social withdrawal	0.76 [0.71–0.80]
Reduced emotional range	0.64 [0.56–0.71]
Alogia	0.71 [0.65–0.77]
Avolition	0.79 [0.75–0.83]
Anhedonia	0.69 [0.62–0.75]

**Table 3:** Intercorrelations between the SNS subscales (Spearman’s rho scores [CI 95%]).

SNS Subscores	Reduced Emotional Range	Alogia	Avolition	Anhedonia
Social withdrawal	0.37 *** [0.25–0.48]	0.48 *** [0.38–0.58]	0.43 *** [0.31–0.54]	0.48 *** [0.37–0.57]
Reduced emotional range		0.42 *** [0.31–0.53]	0.25 *** [0.13–0.37]	0.42 *** [0.30–0.52]
Alogia			0.38 *** [0.27–0.48]	0.51 *** [0.40–0.60]
Avolition				0.52 *** [0.41–0.61]

*** *p* < 0.001.

**Table 4:** Correlational analyses (Spearman’s rho scores [CI 95%]).

	PANSS Total (N = 207)	PANSS Positive	PANSS Negative	PANSS General	HoNOS (N = 187)	GAF (N = 134)	FR (N = 198)	MANSA (N = 182)
SNS total	0.36 *** [0.24–0.48]	0.31 *** [0.19–0.42]	0.33 *** [0.20–0.43]	0.30 *** [0.16–0.42]	0.37 *** [0.21–0.49]	−0.12 [−0.28–0.037]	0.27 *** [0.14–0.39]	−0.40 *** [−0.52–−0.25]
Social withdrawal	0.29 *** [0.15–0.42]	0.31 *** [0.18–0.43]	0.31 *** [0.17–0.43]	0.19 * [0.042–0.32]	0.32 *** [0.16–0.46]	−0.19 * [−0.34–−0.028]	0.27 *** [0.12–0.40]	−0.22 ** [−0.37–−0.075]
Reduced emotional range	0.19 ** [0.053–0.31]	0.18 ** [0.042–0.31]	0.19 ** [0.057–0.31]	0.13 [−0.0078–0.26]	0.18 * [0.045–0.32]	−0.046 [−0.20–0.13]	0.097 [−0.029–0.22]	−0.12 [−0.27–0.028]
Alogia	0.21 ** [0.075–0.33]	0.13 [−0.013–0.26]	0.21 ** [0.081–0.34]	0.20 ** [0.072–0.32]	0.23 ** [0.060–0.37]	−0.015 [−0.18–0.15]	0.12 [−0.0082–0.26]	−0.29 *** [−0.43–−0.16]
Avolition	0.34 *** [0.22–0.46]	0.29 *** [0.17–0.42]	0.25 *** [0.12–0.37]	0.31 *** [0.18–0.43]	0.35 *** [0.21–0.48]	−0.084 [−0.26–0.090]	0.25 *** [0.12–0.37]	−0.46 *** [−0.59–−0.32]
Anhedonia	0.27 *** [0.13–0.40]	0.20 ** [0.066–0.32]	0.22 ** [0.090–0.36]	0.25 *** [0.12–0.38]	0.23 ** [0.074–0.38]	−0.086 [−0.25–0.077]	0.19 ** [0.045–0.32]	−0.36 *** [−0.48–−0.21]
PANSS total	x	0.82 *** [0.75–0.87]	0.78 *** [0.71–0.84]	0.92 *** [0.90–0.94]	0.56 *** [0.44–0.65]	−0.57 *** [−0.69–−0.42]	0.57 *** [0.46–0.66]	−0.43 *** [−0.55–−0.30]
PANSS positive		x	0.53 *** [0.41–0.64]	0.65 *** [0.55–0.75]	0.50 *** [0.37–0.61]	−0.48 *** [−0.62–−0.32]	0.52 *** [0.40–0.63]	−0.34 *** [−0.47–−0.19]
PANSS negative			x	0.61 *** [0.51–0.70]	0.44 *** [0.30–0.56]	−0.43 *** [−0.59–−0.26]	0.53 *** [0.42–0.63]	−0.35 *** [−0.48–−0.20]
PANSS general				x	0.50 *** [0.37–0.61]	−0.53 *** [−0.65–−0.38]	0.49 *** [0.37–0.60]	−0.42 *** [−0.55–−0.29]

* *p* < 0.05; ** *p* < 0.01; *** *p* < 0.001.

**Table 5:** Correlations between SNS and PANSS positive and negative (any item in p1–p7 ≥ 3) (Spearman’s rho scores [CI 95%]).

	PANSS Positive	PANSS Negative
SNS total	0.066 [−0.14–0.26]	0.26 ** [0.063–0.44]
Social withdrawal	0.27 ** [0.090–0.44]	0.31 ** [0.12–0.50]
Reduced emotional range	−0.11 [−0.30–0.087]	0.061 [−0.14–0.27]
Alogia	−0.059 [−0.26–0.16]	0.24 * [0.055–0.41]
Avolition	0.11 [−0.075–0.29]	0.21 * [−0.0038–0.39]
Anhedonia	−0.010 [−0.22–0.19]	0.12 [−0.092–0.32]

* *p* < 0.05; ** *p* < 0.01.

## Data Availability

Under the General Data Protection Regulation (GDPR), our data are considered pseudonymized rather than anonymized and therefore still regarded as personal data. Given that participants have not given informed consent to have their personal data publicly shared, we are legally and ethically not allowed to publish our dataset. Data are therefore only available upon request at the Rob Giel Research center (Data Science Center), due to privacy and ethical reasons.

## Supplementary Materials

The following supporting information can be downloaded at: https://www.mdpi.com/article/10.3390/brainsci15010015/s1, Tabel S1: Missing values of items; Table S2: *p*-values of differences between correlation coefficients in HoNOS; Table S3: *p*-values of differences between correlation coefficients in GAF; Table S4: *p*-values of differences between correlation coefficients in FR; Table S5: *p*-values of differences between correlation coefficients in MANSA.
