# Supplementary material for: Dutch Validation of the Self-Evaluation of Negative Symptoms Scale (SNS)
Source: Brain Sci. 2024 Dec 27;15(1):15. doi: 10.3390/brainsci15010015 (PMC11763429; doi:10.3390/brainsci15010015)
Supplement: Supplementary file 1 [file brainsci-15-00015-s001.zip › brainsci-3385645-supplementary.pdf]

SUPPLEMENTARY MATERIAL

Tabel S1 Missing values of items

Item	Number of missing values
SNS 1	0
SNS 2	1
SNS 3	0
SNS 4	1
SNS 5	5
SNS 6	0
SNS 7	2
SNS 8	0
SNS 9	0
SNS 10	0
SNS 11	1
SNS 12	0
SNS 13	1
SNS 14	0
SNS 15	0
SNS 16	0
SNS 17	0
SNS 18	2
SNS 19	0
SNS 20	1
FR 1	0
FR 2	0
FR 3	0
HoNOS 1	0
HoNOS 2	0
HoNOS 3	0
HoNOS 4	0
HoNOS 5	0
HoNOS 6	0
HoNOS 7	0
HoNOS 8	0
HoNOS 10	0
HoNOS 11	0
HoNOS 14	0
HoNOS 15	0
HoNOS 16	0
MANSA 1	0
MANSA 6	0
MANSA 7	1
MANSA 8	1
MANSA 9	0
MANSA 10	0

MANSA 11	0
MANSA 12	0
MANSA 13	0
MANSA 14	0
MANSA 15	0
MANSA 16	0
MANSA 17	1
MANSA 18	2
MANSA 19	5
MANSA 20	0
PANSS P1	0
PANSS P2	0
PANSS P3	0
PANSS P4	2
PANSS P5	1
PANSS P6	1
PANSS P7	2
PANSS N1	7
PANSS N2	0
PANSS N3	0
PANSS N4	1
PANSS N5	0
PANSS N6	0
PANSS N7	2
PANSS G1	1
PANSS G2	0
PANSS G3	0
PANSS G4	2
PANSS G5	6
PANSS G6	0
PANSS G7	2
PANSS G8	0
PANSS G9	0
PANSS G10	0
PANSS G11	0
PANSS G12	0
PANSS G13	0
PANSS G14	0
PANSS G15	1
PANSS G16	2

Table S2 P-values of differences between correlation coefficients in HoNOS

**HONOS**	Reduced emotional range	Alogia	Avolition	Anhedonia
Social withdrawal	0.045*	0.10	0.34	0.11
Reduced emotional range		0.26	0.026*	0.26
Alogia			0.059	0.51
Avolition				0.044*

*p <0.05; ** p<0.01; ***p<0.001

Table S3 P-values of differences between correlation coefficients in GAF

**GAF**	Reduced emotional range	Alogia	Avolition	Anhedonia
Social withdrawal	0.073	0.023*	0.12	0.12
Reduced emotional range		0.38	0.36	0.34
Alogia			0.22	0.2
Avolition				0.49

*p <0.05; ** p<0.01; ***p<0.001

Table S4 P-values of differences between correlation coefficients in FR

**FR**	Reduced emotional range	Alogia	Avolition	Anhedonia
Social withdrawal	0.016*	0.016*	0.39	0.13
Reduced emotional range		0.38	0.044*	0.118
Alogia			0.050	0.16
Avolition				0.20

*p <0.05; ** p<0.01; ***p<0.001

Table S5 P-values of differences between correlation coefficients in MANSA

**MANSA**	Reduced emotional range	Alogia	Avolition	Anhedonia
Social withdrawal	0.11	0.16	<0.001***	0.025*
Reduced emotional range		0.013*	<0.001***	0.0010**
Alogia			0.0080**	0.14
Avolition				0.059

*p <0.05; ** p<0.01; ***p<0.001
